# Producer and Veterinarian Perspectives towards Pain Management Practices in the US Cattle Industry

**DOI:** 10.3390/ani11010209

**Published:** 2021-01-16

**Authors:** Ivelisse Robles, Andreia G. Arruda, Emma Nixon, Elizabeth Johnstone, Brooklyn Wagner, Lily Edwards-Callaway, Ronald Baynes, Johann Coetzee, Monique Pairis-Garcia

**Affiliations:** 1 Department of Population Health and Pathobiology, College of Veterinary Medicine, North Carolina State University, Raleigh, NC 27606, USA; irobles@ncsu.edu (I.R.); enixon@ncsu.edu (E.N.); bwagner2@ncsu.edu (B.W.); Ronald_Baynes@ncsu.edu (R.B.); 2 Department of Veterinary Preventive Medicine, College of Veterinary Medicine, The Ohio State University, Columbus, OH 43210, USA; arruda.13@osu.edu; 3 Department of Animal Sciences, Colorado State University, Fort Collins, CO 80521, USA; Elizabeth.Johnstone@colostate.edu (E.J.); Lily.Edwards-Callaway@colostate.edu (L.E.-C.); 4 Department of Anatomy and Physiology, College of Veterinary Medicine, Kansas State University, Manhattan, KS 66506, USA; jcoetzee@vet.k-state.edu

**Keywords:** cattle, animal welfare, pain mitigation, analgesics, attitudes, survey, FARAD

## Abstract

**Simple Summary:**

A key aspect to improve animal welfare is to reduce pain experienced by the animal. However, pain management practices have not been widely adopted in the United States (US) cattle industry. Furthermore, for a veterinarian or producer to relieve pain in cattle, analgesics must be provided in an extra-label drug manner. Currently, research describing pain mitigation strategies used by cattle producers and the study of barriers to implement those strategies from a producer or veterinarian perspective is limited. Understanding challenges faced by these stakeholders is key in improving on-farm pain management strategies. Therefore, the objectives of this study were to explore producer and veterinarian perspectives on pain. Albeit analgesia use increased in the past ten years for some producers and the majority of veterinarians, administering analgesics for pain management on US cattle farms remains a challenge. From a producer perspective, drug cost, availability and logistics for administration. From a veterinarian perspective, lack of Food and Drug Administration (FDA) products hinders the support of on-farm protocols requiring extra-label drug use. Future steps to improve analgesic use on-farm include identifying and approving drugs that demonstrate efficacy for managing pain and disseminating educational resources to support stakeholders in both the implementation and drug withdrawal process.

**Abstract:**

Producers and veterinarians are considered responsible for improving animal welfare, as they are responsible for implementing practices that directly impact the animal’s well-being. Most husbandry procedures performed in cattle do not include pain mitigation, and understanding challenges faced by these stakeholders to use analgesics is key in improving on-farm pain management strategies. Therefore, the objectives of this study were to explore producer and veterinarian perspectives on pain management practices by (1) exploring inquires received by Food Animal Residue Avoidance Databank (FARAD) regarding analgesic use in cattle and (2) using a survey instrument to identify factors that impact pain management implementation in the US cattle industry. Albeit analgesia use increased in the past ten years for some producers and the majority of veterinarians, administering analgesics for pain management on US cattle farms remains a challenge. From a producer perspective, drug cost, availability and logistics for administration. From a veterinarian perspective, lack of Food and Drug Administration (FDA) products hinders the support of on-farm protocols requiring extra-label drug use. Future steps to improve analgesic use on-farm include identifying and approving drugs that demonstrate efficacy for managing pain in cattle and disseminating educational resources to support stakeholders in both the implementation and drug withdrawal process.

## 1. Introduction

Public scrutiny has increased over the past decade regarding the raising of livestock species, with an emphasis on improving individual animal welfare through changes to daily management practices [1]. A key aspect in the conversation to improve animal welfare is to reduce pain experienced by the animal [2,3,4]. It is well documented that cattle experience pain as a consequence of common procedures performed routinely on-farm such as castration [5,6,7], disbudding and dehorning [8,9]. These procedures result in short-term deviations to the animal’s physiology, behavior and productivity, and there is recent work in disbudding which suggests there may be longer term impacts of these procedures when pain is left unmitigated [10,11]. To manage this pain, local anesthetics and analgesics such as non-steroidal anti-inflammatory drugs (NSAIDs) have been identified in the literature as options to mitigate post-procedural pain sensitivity [12].

However, pain management practices have not been widely adopted for use in the United States (US) cattle industry [13,14]. This is most likely due to the absence of US Food and Drug Administration (FDA; regulatory agency in the US responsible for assessing drug safety and efficacy) label-approved drugs specific for the control of procedural pain in cattle. To date, transdermal flunixin remains the only approved NSAID for pain management in cattle and its use is limited to only treating pain associated with foot rot [15]. Thus, in order for a veterinarian or producer to relieve pain in cattle, analgesics must be administered as extra-label drug use (ELDU) at the discretion of the veterinarian [16]. Veterinarians must then rely on working with programs such as the Food Animal Residue Avoidance Databank (FARAD), a United States Department of Agriculture (USDA) sponsored project that provides scientifically based withdrawal interval recommendations for ELDU in food animals.

Currently, there is very limited research available describing pain mitigation strategies used by cattle producers in the US [12,14,17,18]. Furthermore, minimal work to date has explored the barriers to implementing pain management in cattle from a producer or veterinarian perspective [4,16]. Understanding the challenges that producers and veterinarians are confronted with is key to improving on-farm pain management strategies for the US cattle industry. Therefore, the objectives of this study were to explore producer and veterinarian perspectives on pain management practices by (1) exploring inquires received by FARAD regarding analgesic use in cattle and (2) using a survey instrument to identify factors that impact pain management implementation in the US cattle industry.

## 2. Materials and Methods

### 2.1. Food Animal Residue Avoidance Databank Data Description

Data from FARAD regarding the number and nature of inquires received regarding flunixin/flunixin meglumine, meloxicam and acetylsalicylic acid (aspirin) use in cattle through 2015–2019 were extracted by one of the co-authors (EN). The FARAD is a national, cooperative, USDA-sponsored project that provides scientifically based withdrawal interval recommendations for ELDU in food animals. There are five FARAD centers located in the US (North Carolina State University, University of California, University of Florida, Kansas State University, and Virginia Polytechnic Institute and State University). Inquiries regarding drug withdrawal information were submitted via telephone (1-888-873-2723) or online (http://farad.org/contact-us.html) by veterinarians. Information was collected and includes contact (email address, phone number), case (species, numbers of animals, average body weight, food product), and drug information (dose and route). All inquiries are responded to within 72 h. Although this program is provided as a service to veterinarians requiring withdrawal information on ELDU, it is important to remember that these data are not necessarily indicative of actual drug administration as these inquiries may be hypothetical in nature.

### 2.2. Survey

#### 2.2.1. Development and Implementation

Survey questions regarding veterinarian and producer perspectives on pain mitigation practices in cattle are a subset of data collected from an Institutional Review Board-approved survey developed by Colorado State University (CSU) in partnership with Kansas State University (CSU IRB #18-7937H; Johnstone et al., in press). The survey was an adaptation from a 2017 UK survey [19] and was administered via Qualtrics survey software (Qualtrics, Provo, Utah, US) by CSU and Informa-Engage Research between 11 June to 10 August 2018. Responses to all survey questions were anonymous and no individual identifying information was associated with the responses.

#### 2.2.2. Population

The survey targeted veterinarians and producers who treat and raise, respectively, dairy and/or beef cattle in the US. Listservs were used to distribute the survey and included Farm Progress (*n* = 34,681), American Association of Bovine Practitioners (*n* = 3628), Academy of Veterinary Consultants (*n* = 901), National Milk Producers Federation Farm Evaluators (*n* = 643), Dairy Moms Facebook group (*n* = 1797), and Dairy Girl Network Facebook group (*n* = 4927). Each listserv received an initial invitation and two reminder emails approximately one week apart of each other. The population for this survey included a final number of 1066 respondents.

#### 2.2.3. Survey Questions

The survey had a total of eleven questions including four demographic questions (Survey S1). Demographic questions included gender, age, role within cattle industry (veterinarian or producer) and location of operation. In addition to demographic questions, seven questions were selected with the aim to explore aspects of producer and veterinarian perspectives on pain management practices and to describe factors associated with pain management choice (Survey S1). Questions used “branch logic” to indicate role (veterinarian or producer) and would generate questions specific to a veterinarian or a producer, based on the role selected. Depending on the response of the participant, the survey included or excluded specific questions. For example, respondents were asked to classify changes to analgesic use over the last ten years (increased used, stayed the same, decreased use). If respondents increased their analgesic use, those respondents, and only those respondents, were asked to select from eleven statements specific to analgesic use increase. Likewise, if respondents decreased their analgesic use, those respondents, and only those respondents, were asked to select from seven statements specific to analgesic use decrease (Survey S1). Respondents were also asked to respond (Yes/No) if they considered that their knowledge about recognizing and treating pain in adult cattle is adequate. Respondents were asked to select from ten pain relief drugs (analgesics) they had knowledge of and feel comfortable using in their operation or practice.

For the question regarding specific factors that impact a respondent’s decision on pain management use in cattle, a Likert scale format was utilized (1-Not at all important, 2-slightly important, 3-moderately important, 4-very important, 5-extremely important).

### 2.3. Statistical Analyses

Data from FARAD inquiries were summarized as counts at the drug (flunixin, meloxicam and aspirin) and year (2015–2019) level from the database and input into a spreadsheet (Microsoft Excel 2016; Microsoft Corp., Redmond, WA, USA). All analyses were conducted using Stata 14.2 (College Station, TX, USA). A multivariable generalized linear model (GLM) specifying the Poisson family was used to investigate the association between the three above-mentioned drugs and year as predictors (independent variables) for the counts of inquires (dependent variable). Model building followed a forward stepwise approach, and model fit was verified by calculating and plotting the Anscombe residuals, which should be normally distributed.

Survey responses were compiled into another spreadsheet, and recording errors were removed and recorded as missing. Basic descriptive statistics were calculated for captured responses. Binary and categorical variables/questions (e.g., sex, location, and age category) were described using frequency tables (%), and continuous variables/questions (e.g., Likert scale questions) were described using means (±standard deviation, SD). A multivariable logistic regression model was built using a forward stepwise approach to describe the association between having reported an increase in the use of analgesics in the past ten years (dependent variable) and the respondent’s role (veterinarian or producer). Respondent’s age category and sex were accounted for in the model as covariates, and retained in the final model if *p* < 0.05 and/or if they changed the other model coefficients by more than 20% [20].

Within the subset of survey respondents that reported having increased the use of analgesics within the past ten years (this corresponded to the vast majority of respondents), the same logistic model approach described above was used to investigate the association between the respondent’s role (veterinarian or producer) on selecting each of the eleven reasons (Survey S1). As such, a model was built for each possible reason as an outcome, and sex and age category were accounted for when appropriate.

Finally, in order to understand the importance of eleven different potential reasons (Survey S1) on the decision for use of analgesic drugs in cattle, Likert scale-based answers were summarized separately by respondent’s role (veterinarian or producer). Non-parametric Mann-Whitney test was used to compare the numerical values from Likert scale-based answers between the two groups (producer versus veterinarian) for each potential reason for using analgesic drugs separately. Statistical significance for all tests and models was declared at *p* < 0.05, a tendency was declared at 0.05 ≤ *p* < 0.10. Although there is the chance that potential respondents were on multiple listservs, we feel confident that the chances of duplicate responses is extremely low and analysis was performed under that assumption.

## 3. Results

### 3.1. Inquiries to FARAD

**Figure 1:**
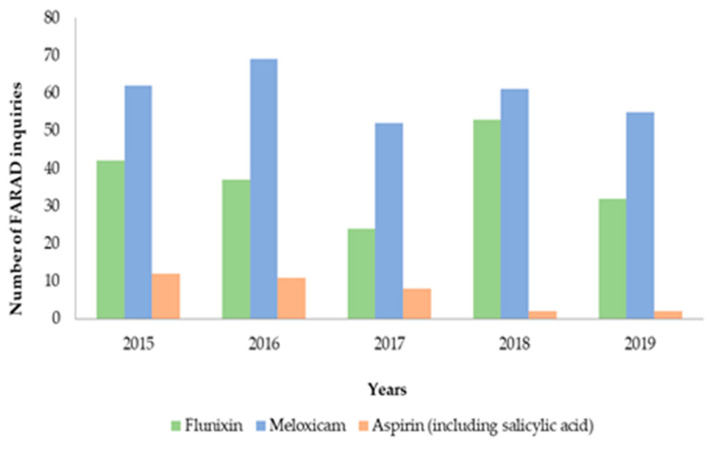
Food Animal Residue Avoidance Databank (FARAD) cattle inquiry number for flunixin (*n* = 187), meloxicam (*n* = 299), and aspirin (*n* = 34) for year 2015 to 2019.

In total, FARAD received 520 inquiries specific to cattle, for analgesics meloxicam, flunixin, and aspirin from 2015-2019. These inquiries represented approximately 2.98% of all inquiries received by FARAD on an annual basis (3485/yr in the last 5 yr). Meloxicam was noted as the analgesic with the greatest number of inquiries (*n* = 299), followed by flunixin (*n* = 187) and aspirin (*n* = 34). Total number of inquiries are presented in Figure 1. Results from the final multivariable model indicate that both flunixin (incidence rate ratio (IRR) = 5.37; 95% confidence interval (CI): 3.74–7.70) and meloxicam (IRR = 8.54; 95% CI: 6.02–12.12) had higher incidence rates of inquires compared to aspirin (*p* < 0.001). When evaluating FARAD inquiries by year, a 0.72 IRR decrease (95% CI: 0.55–0.96) in 2017 was reported compared to 2015 (*p* = 0.02). There was also a tendency for the incidence rate to decrease in 2019 compared to 2015 (IRR = 0.77; 95% CI: 0.58–1.01; *p* = 0.06). Ascombe residuals were normally distributed, indicating good model fit.

For the majority of FARAD inquiries across all three analgesics, a purpose for the inquiry was not provided (76.5% aspirin; 71.1% flunixin; 68.0% meloxicam inquiries with no purpose). For the meloxicam inquiries that listed a purpose (*n* = 97), disbudding/dehorning (22.7%), general pain (17.5%), and lameness (14.4%) were most commonly noted. For those flunixin inquiries that listed a purpose (*n* = 54), accidental-injury (13.0%), mastitis (11.1%), and lameness (9.3%) were noted. For aspirin (*n* = 8), the only purpose that was listed more than once was calving (37.5%). Administration route listed also varied per analgesic. The most commonly listed administration route for aspirin and meloxicam was oral (96.3%; 97.1%, respectively) while flunixin had several listed administration routes, which included intramuscular (28.3%), intravenous (27.3%), topical (14.0%) and oral (12.3%).

### 3.2. Survey

A total of 46,577 surveys were sent electronically with a response rate of 3.8% (1790 surveys). From those surveys, 1222 were 80% complete. Surveys in which respondents were not in role of producer or veterinarian were outside of the scope of this study for response comparisons and were removed from analysis (156 surveys). In total there were 1,066 survey responses included in the results. Demographic responses for producers and veterinarians are presented in Table 1. Of all the respondents, 497 (46.6%) were producers and 569 (53.4%) were veterinarians. The majority of respondents identified as males (80.3% producers and 63.4% veterinarians). Regarding respondent age, 50.9% of producer respondents selected the 51 to 70 year old age category with the least number of producer respondents selecting the 21 to 30 year old age category (6.6%). Conversely, about 45% of veterinarian respondents selected the 21 to 40 year old age category and about 35% selected the 41 and 60 year old age category. The greater than 70 age category was least represented for veterinarians. All US regions were represented in both roles (producer, veterinarian). The Midwest region was the most represented for both producers and veterinarians while the Northeast region was least represented for producers and the Southeast region for veterinarians (Table 1).

A total of 70.1% of the producers and 68.7% of the veterinarians considered themselves knowledgeable about recognizing and treating pain in cattle. There was no difference about perception of knowledge on recognizing and treating pain in cattle between veterinarians and producers (*p* = 0.30), but an age category effect was observed. Respondents from the oldest age category (>70 years old) had lower odds (Odds Ratio (OR) = 0.29; 95% CI: 0.16–0.51) of reporting considering themselves knowledgeable when compared to the 41–50 years old age category (*p* < 0.01).

Survey responses on pain drugs that participants had knowledge and feel comfortable using are presented in Table 2. When ranked within role from all options selected, the majority of producer respondents selected flunixin (e.g., Banamine^®®^, Madison, NJ, USA; 63.4%), lidocaine (51.9%) and aspirin (48.1%) as an analgesic that they have knowledge of and feel comfortable using while the majority of veterinarian respondents selected flunixin (99.5%), lidocaine (99.3%), oral meloxicam (80.5%), and aspirin (56.1%).

For the option “Other” veterinarian responses included butorphanol (*n* = 19), gabapentin (*n* = 5), dexamethasone (*n* = 3), morphine (*n* = 3) combinations like xylazine and morphine, and epidural (*n* = 3). Not all respondents that selected this option included a response and some did not provide a pain drug in the response. One veterinarian wrote “I have meloxicam, aspirin, and phenylbutazone, but am not experienced/comfortable using them regularly in food animals”. Producers responses for this Other drug category included dexamethasone (*n* = 4).

When ranked within role from all options selected, both producers (63.1%) and veterinarian (39.0%) respondents selected “personal experience” as the main source from where they ‘feel that they have obtained most of the knowledge about treating and recognizing pain’. Producer and veterinarian respondents also frequently selected ‘journals/articles’ (12.6% producers, 11.4% veterinarians) and “continuing education” (11.0% producers, 35.0% veterinarians) as their main source of knowledge about treating and recognizing pain.

When modeling the impact of respondent’s role in selecting increased analgesic use in the past ten years, producers had lower odds of having reported an increase in use compared to veterinarians (OR = 0.12; 95% CI = 0.09–0.16; *p* < 0.001), after accounting for age and sex. The majority of the veterinarian respondents (76.5%) reported an increase in analgesic use while a little over a third of producer respondents (32.2%) reported an increase in analgesic use over the last ten years. Over half of the producer respondents (58.5%) reported that their analgesic use stayed the same, while only 22.2% of the veterinarian respondents reported that their analgesic use stayed the same. Less than 10.0% of the producer respondents (9.3%) and 1.4% of the veterinarian respondents decreased analgesic use in the past ten years. Respondent’s reasons for analgesic use increased in the past ten years are presented in Table 3. Both producer (65.4%) and veterinarian respondents (84.1%) selected “change of attitude” as the main reason for increased analgesic use in the past ten years. Change in practice or operation protocols was reported frequently for producers (62.8%) while 67.8% of veterinarians increased analgesic use based on “new evidence for analgesic effectiveness”. The majority of producer (57.1%) and veterinarian respondents (66.7%) also believed that analgesic administration improved cattle health and performance.

Results from these logistic models exploring the reasons for increased analgesic use in the past ten years revealed that producers had lower odds of selecting the reason ‘analgesic effectiveness’ (OR = 0.52; 95% CI: 0.36–0.76; *P* = 0.001), ‘decreased prices’ (OR = 0.38; 95% CI: 0.23–0.60; *p* < 0.001), “change in perception of pain” (OR = 0.50; 95% CI: 0.34–0.72; *p* < 0.001), ‘change of attitude’ (OR = 0.32; 95% CI: 0.21–0.49 *p* < 0.001), ‘peer influence’ (OR = 0.32; 95% CI: 0.21–0.48; *p* < 0.001), “maintain consumer confidence” (OR = 0.52; 95% CI: 0.36–0.75; *p* = 0.001), and “cattle improved health and performance” (OR = 0.67, 95% CI: 0.46–0.97; *p* = 0.036), when compared to veterinarians selection. Sex and age category were accounted for in all models.

Reasons given by producer and veterinarian respondents for analgesic use decrease in the past ten years are presented in Table 4. When ranked within role from all options selected, both producers and veterinarian respondents (42.2 and 50.0%, respectively) selected that “I am not comfortable using an analgesic unless it has been approved by FDA” as one of the most common reasons for decreased analgesic use. Veterinarian respondents also frequently selected “inconvenience to administer the drug” (50%) and “it was not lasting long enough after one dose” (50%) as reasons to decrease analgesic use (Table 4). Analgesic cost was the second most commonly selected response by producers (35.6%).

Summary from Likert scale-based responses are presented in Table 5. Mann-Whitney tests revealed differences on the following factors when comparing producers to veterinarian responses: “duration of pain control/analgesic effect of drug”, “short withhold period”, “animal’s ability to feel pain”, “improving safety of the caregiver/operator, and ‘how painful I consider the procedure to be” (*p* ≤ 0.01). Full Likert scale distribution of responses are presented in Table 6.

## 4. Discussion

The responsibility of improving animal welfare is directly placed on producers and veterinarians [21], as they are responsible for implementing practices that ultimately impact the animal’s well-being under their care [4]. An important aspect of animal welfare is eliminating or mitigating pain experienced as a consequence of production practices. Currently in US, most husbandry procedures performed in cattle do not include pain mitigation [14,17] and there is limited information available evaluating the barriers to implementing pain management in cattle [4]. Therefore, the objectives of this study were to explore producer and veterinarian perspectives on pain management practices by (1) exploring inquires received by FARAD regarding analgesic use in cattle and (2) identifying factors that impact pain management implementation in the US cattle industry for both producers and veterinarians.

Results from these surveys demonstrate that FARAD inquiries for cattle specific to meloxicam, flunixin and aspirin generated a little over 100 inquiries each year. These inquiries represented approximately 2.98% of all inquiries received by FARAD on an annual basis (3485/yr in the last 5 yr). This is in contrast with work conducted by Wang et al. 2003 [22] that identified inquiries specific to NSAIDs for all species represented approximately 12.5% of calls over a three-year period and identified that close to half (48.0%) of all the inquiries were specific to cattle over the same time period. Additionally, the present work indicates that inquiries specific to these NSAIDs in cattle during this time period (2015–2019 were or tended to be lesser when compared with previous years. When evaluating this category of drugs alone, the results from the present study are in contrast to work conducted by Riviere and colleagues in 2017 [23] that suggested that overall FARAD inquiry numbers will increase by double digits. Although inquiry interest varied by analgesic, the overall decrease may be due to better access to drug information through peer-reviewed publications and continuing education. Since 2015, over 40 research studies have been published regarding analgesic efficacy of meloxicam, flunixin and aspirin in processing procedures specific to cattle. In addition, opportunities to learn about pain management in cattle have increased for both veterinarians and producers since 2015. This has included conferences such as the Beef Cattle Welfare Symposium and Dairy Welfare Symposium that specifically addressed pain management as well as partnerships with extension programs and popular press magazines such as Hoard’s dairymen and Drovers that have highlighted pain management [24,25]. Given veterinarians and producers both noted journal articles and continuing education as two sources for which they obtained most of their knowledge about treating and recognizing pain, it is possible that these individuals are gaining access to information specific to these drugs, thus decreasing total inquiries received by FARAD. Additional possibilities for a decrease in FARAD inquiries may be associated with drug access and availability. In 2016 [26] and 2019 [27], shortages for flunixin meglumine and meloxicam were noted due to manufacturing delays. This may have discouraged veterinarian inquiries if the drug was not accessible for use.

It is unlikely that inquiries decreased because the purpose for the drug was no longer needed. Although the majority of inquiries did not list a purpose, for those that did, the most commonly noted reasons for analgesic use are still current issues in the US cattle industry to date. For example, clinical mastitis and lameness were noted as the secondary and tertiary purposes listed for meloxicam and flunixin use. According to the 2014 USDA report on health and management practices on US dairy operations, mastitis and lameness were reported in 25% and 17% of cows [28]. Additionally, when assessing US dairy farms alone, over 90% of farms still disbud calves [28]. This procedure was cited as the primary purpose listed for meloxicam use in FARAD inquiries and would continue to be an area of interest given there has been an increased expectation to manage disbudding pain by marketing chains and retailers [29]. In contrast, the US Beef industry may have decreased overall disbudding/dehorning events given an increase in popularity of polled genetics, thus eliminating the need for the procedure [16]. Similar initiatives are under consideration in the dairy industry [30]. However, incorporating polled genetics into dairy cattle populations in the US has been very challenging due tradeoffs in genetic merit [30]. Thus, it is unlikely that FARAD inquiries decrease is driven by polled genetics or disease occurrence on farm as neither have had a dramatic shift in the industry.

Results from the survey portion of this study aimed to explore aspects of producer and veterinarian perspectives on pain management practices and to describe factors associated with pain management choices. Response rate was lower than other studies that have targeted only veterinarian respondents [13,14]. Several factors could have contributed to the low response rate. These factors potentially include method of survey dissemination, sources used to distribute the survey, type of distribution electronically rather than paper and the interest of individuals in the roles studied to respond and to complete the questions included in the survey. Another limitation of this research is the potential for bias in different aspects. There was not an even representation of respondents based on location. Also, the use of a quantitative method to explore barriers and challenges in an area that has had limited previous work to guide the development of the survey could have limited the findings. Nevertheless, number of respondents in the roles of veterinarians and producers and responses obtained in this research can provide valuable insight in the scope researched.

Producers and veterinarians were not different regarding their perceived knowledge and comfort in recognizing and treating pain in cattle. These findings are similar to work conducted by Johnstone and colleagues [16] and suggests both producers and veterinarians believe that managing pain in cattle is important. In addition to individual knowledge of a topic, attitudes of those that work directly with animals can play a major role in the ability to improve animal care, treatment and overall animal welfare. For example, research specific to timely euthanasia on-farm has demonstrated that individuals working with animals that show empathetic and confident personality traits are more likely to successfully perform euthanasia in a timely and humane manner [31,32].

Given that an overwhelming majority of producers and veterinarians whom responded to this survey feel confident in recognizing and treating pain in cattle, the US cattle industry may move more quickly towards implementing effective strategies to control pain once a culture of pain management is established. Thus, in the context of this survey, respondents can indeed take action in managing pain on-farm given they are confident in their knowledge and skills and can then make long-term changes to the culture of the industry as a whole [33].

The survey results also demonstrated an increase in analgesic use in 76.4% of veterinarian respondents over the past ten years. These results support the concept that there has been a shift in the culture and attitude of US cattle veterinarians regarding pain management. Our results are in agreement with work conducted in the United Kingdom regarding attitudes towards pain management by veterinarians [19] and suggest shared interest in managing pain for cattle on a global scale for the veterinary community [4].

Nevertheless, changes to analgesic use by veterinarians may not result in large scale actionable changes in pain management on-farm [34] given only 32.2% of producers have increased analgesic use over the past ten years. Although veterinarians are viewed by producers as valuable resources in regards to animal health and welfare, the producer most often performs daily decision-making specific to animal care and treatment [35]. Therefore, the willingness of the producer to implement pain management protocols will likely have a greater impact on individual animal welfare. Designating analgesic drug use as requiring veterinary oversight may also serve as a barrier to the widespread adoption of analgesic use on farms. Identifying analgesic drug formulations that can be distributed over-the-counter will likely increase adoption of analgesic drug use on farms in the future.

Despite producers valuing the recommendations of their veterinarians (moderately to extremely important factor for producers) and their belief in managing pain, producers, and some veterinarians, do not feel ‘comfortable using an analgesic unless it has been approved by FDA’. Given there is only one FDA-approved drug available for pain management in cattle in the US, this barrier may have significant and long-term impacts on improving pain management on farm. Furthermore, the restricting and ambiguous guidelines with respect to pain mitigation can discourage its use. Recommendations of associations compared to what is allowed in the extra label use could create a setback for veterinarians in administering analgesics. These results are in agreement with Johnstone and colleagues [16] that noted almost 90% of US veterinarians participating in their survey agreed that regulatory agencies including the FDA limit their ability to use analgesics in cattle. This issue is not unique to cattle veterinarians in the US alone. Wagner and colleagues [35] conducted a similar study assessing the perspective of swine veterinarians on implementing pain management for piglets and identified that the lack of approved products validated for efficacy is one of the main concerns to implementing pain management on swine farms.

Albeit analgesia use increased in the past ten years for some producers and the majority of veterinarians, both face challenges implementing pain mitigation strategies. Demonstrated efficacy for managing pain in dairy cattle must be achieved to obtain FDA approval. An approved drug must not only demonstrate efficacy for managing pain but must also be cost effective and easy to administer. Producers selected cost as the second most important factor for decreasing use of analgesics over the past ten years and both producers (77.5%) and veterinarians (83.5%) cited cost as a moderate to extremely important factor influencing the decision to use an analgesic. In addition, 85 and 90% of producers and veterinarians identified “ease of administration” as a moderate to extremely important factor affecting the decision to use an analgesic. Therefore, if a drug is expensive or is difficult to administer, it will discourage stakeholders from using it despite having an FDA approval for pain management. Thus, efforts must include examining potential ways to address challenges related to ease of administration. Better understanding challenges faced by producers with integration of both quantitative and qualitative approaches will help guide the path to continue the efforts to support stakeholders directly responsible for the care of animals. Offering educational resources and support for both veterinarians and producers is imperative to increase their confidence with respect to analgesics use. Programs like FARAD will continue to serve an important role in the livestock community by providing guidance on analgesic use for pain management as a means to improve on-farm animal welfare.

## 5. Conclusions

In conclusion, a majority of producers and veterinarians who responded to this survey feel confident in recognizing and treating pain in cattle. Producers and veterinarians in US have increased the use of analgesia to mitigate pain in cattle. Albeit. analgesia use increased in the past ten years for some producers and the majority of veterinarians, both face challenges implementing pain mitigation strategies. From a producer perspective, drug cost, availability, and logistics for administration. From a veterinarian perspective, lack of FDA products hinders the support of on-farm protocols requiring extra-label drug use. Producers and veterinarians benefit from educational resources and continued support from programs like FARAD to improve animal welfare through pain mitigation. Continued efforts to improve animal welfare must also include FDA drug approval with demonstrated efficacy for managing pain in cattle that is cost effective and easy to administer.

## Figures and Tables

**Table 1:** Demographics summary of all responders (*n* = 1066).

		Role	
Factor		Producer (*n* = 497)	Veterinarian (*n* = 569)
Gender	Male Female No Response *	80.3% 19.5% 0.2%	63.4% 36.2% 0.3%
Age (years)	21 to 30 31 to 40 41 to 50 51 to 60 61 to 70 >70 No Response	6.6% 16.3% 14.1% 26.6% 24.3% 11.5% 0.6%	17.8% 27.1% 16.9% 17.9% 17.2% 3.0% 0.8%
Location or Operation or Practice by Region †	West Southwest Midwest Southeast Northeast No Response	17.3% 14.3% 42.1% 16.9% 9.1% 0.4%	14.8% 17.4% 52.2% 10.9% 14.6% 0.8%

* No Response= individuals that did not respond to the question. † West: WA, OR, CA, NV, UT, ID, MT, WY, CO; Southwest: AZ, NM, TX, OK; Midwest: ND, SD, NE, KS, MN, IA, MOWI, IL, IN, MI, OH; Southeast: AR, LA, MS, AL, TN, KY, GA, FL, SC, NC, WV, VA, DC, MD, DE; Northeast: NJ, PA, RI, CT, NY, MA, VT, N, ME.

**Table 2:** Survey respondents (*n* = 1066) on pain drugs that they have knowledge and feel comfortable using based on role *.

	Producer	Veterinarian
	(*n* = 481)	(*n* = 568)
Lidocaine	51.9% (258)	99.3% (565)
Oral Meloxicam	16.1% (80)	80.5% (458)
Meloxicam Injection (Metacam^®®^ Injection)	8.2% (41)	11.2% (64)
Flunixin (e.g., Banamine^®®^) Injection	63.4% (315)	99.5% (566)
Flunixin (e.g., Banamine^®®^) Pour-on	11.3% (56)	45.0% (256)
Aspirin	48.1% (239)	56.1% (319)
Phenylbutazone	13.3% (66)	18.6% (106)
Ketoprofen (Anafen^®®^ Injection)	4.6% (23)	6.9% (39)
Other (please specify)	2.0% (10)	7.4% (42) ^1^
None of these	15.7% (78)	0.0% (0)

* Respondents had the option to select all that apply therefore the total of each column does not add to 100%; ^1^ Food and Drug Administration.

**Table 3:** Survey respondent (*n* = 591) reasons for increased analgesic use in the last 10 years based on role *.

	Producer	Veterinarian
	(*n* = 156)	(*n* = 435)
New evidence of analgesic effectiveness	52.6% (82)	67.8% (295)
Requirement of a quality assurance program	24.4% (38)	21.1% (92)
Decreased prices for analgesics	16.0% (25)	33.6% (146)
Change in your perception of pain in cattle	49.4% (77)	65.5% (285)
Changing farmer or veterinarian attitudes	65.4% (102)	84.1% (366)
Change in practice or operation protocols	62.8% (98)	57.5% (250)
Influence from colleagues/fellow producers	24.4% (38)	50.1% (218)
Mandated by a retailer or packer	7.7% (12)	6.0% (26)
Maintain consumer confidence in livestock production practices	45.5% (71)	60.9% (265)
Cattle that receive analgesia look better than cattle that don’t	26.3% (41)	51.7% (225)
Cattle that receive analgesia have improved health and performance	57.1% (89)	66.7% (290)

* Respondents had the option to select all that apply therefore the total of each column does not add to 100%. Although dates were not provided in the question the timeframe represented was 2008 to 2018.

**Table 4:** Survey respondent (*n* = 53) reasons for decreased analgesic use in the last 10 years based on role *.

	Producer	Veterinarian
	(*n* = 45)	(*n* = 8)
Currently available analgesic drugs are not effective at reducing pain	2.2% (1)	12.5% (1)
Currently available analgesic drugs are inconvenient to administer	20.0% (9)	50.0% (4)
Currently available analgesic drugs do not last long enough after 1 dose to justify their use	26.7% (12)	50.0% (4)
Currently available analgesic drugs are too expensive	35.6% (16)	12.5% (1)
I do not know the meat and milk withhold periods for the analgesic drugs	15.6% (7)	0.0% (0)
Currently available drugs do not improve health and performance	26.7% (12)	25.0% (2)
I am not comfortable using an analgesic unless it has been approved by FDA ^1^	42.2% (19)	50.0% (4)

* Respondents had the option to select all that apply therefore the total of each column does not add to 100%.; ^1^ Food and Drug Administration. Although dates were not provided in the question the timeframe represented was 2008 to 2018.

**Table 5:** Survey respondent factors (*n* = 1066) impacting the decision to use analgesics drug in adult cattle and calves based on role.

Factor	Not at All Important		Slightly Important		Moderately Important		Very Important		Extremely Important		*n* = ^1^	
	P ^3^	V ^4^	P	V	P	V	P	V	P	V	P	V
FDA approval status ^2^	7.4%	2.5%	15.9%	15.0%	18.1%	31.8%	38.2%	31.6%	20.4%	19.1%	471	568
Cost	6.0%	2.4%	16.6%	14.0%	36.0%	40.0%	29.8%	32.9%	11.7%	10.6%	470	567
Recommendation of Veterinarian (Producers only)	2.3%		5.3%		18.6%		47.5%		26.3%		472	0
Duration of pain control/analgesic effect of drug	3.4%	0.0%	10.9%	3.2%	34.0%	26.9%	43.6%	53.3%	8.1%	16.6%	468	567
Ease of administration	3.4%	0.7%	11.7%	10.1%	28.8%	31.9%	39.9%	42.8%	16.2%	14.5%	469	566
Short withhold period	7.7%	1.9%	19.7%	9.9%	30.4%	32.8%	27.6%	38.3%	14.6%	17.1%	467	568
Animal’s ability to feel pain	1.7%	0.7%	9.6%	3.2%	30.6%	23.3%	44.5%	50.1%	13.6%	22.6%	470	569
Improving safety of the caregiver/operator	4.1%	1.6%	14.1%	10.1%	26.1%	25.3%	39.1%	45.1%	16.7%	17.9%	468	568
Improved production outcomes	2.6%	1.8%	9.4%	8.3%	24.0%	25.8%	45.1%	47.4%	18.9%	16.8%	466	567
How painful I consider the procedure to be	4.5%	0.4%	11.1%	4.2%	29.5%	19.2%	43.6%	52.4%	11.3%	23.8%	468	569
Time of onset of drug activity	4.3%	2.3%	11.6%	11.3%	36.1%	34.7%	39.4%	40.6%	8.6%	11.1%	465	569
Request of producer (Veterinarians only)		3.5%		15.9%		36.9%		31.6%		12.0%	0	568

^1^ Number of respondents for each factor; ^2^ Food and Drug Administration; ^3^ V = veterinarian; ^4^ P = producer.

**Table 6:** Summary (mean ± standard deviation) and Mann-Whitney test results (*p*-value) for Likert scale-based factors ranked by producers and veterinarians for each of the items below corresponding to their importance in impacting the decision to use an analgesic drug in adult cattle and calves (1: minimum/not at all important − 5: maximum/extremely important).

Factor	Producer		Veterinarian		*p*-Value ^1^
	*n* = ^2^	Mean ± SD	*n* =	Mean ± SD	
FDA approval status ^3^	471	3.5 ± 1.19	568	3.5 ± 1.04	0.59
Cost	470	3.2 ± 1.05	567	3.4 ± 0.93	0.15
Recommendation of Veterinarian (Producers only)	472	3.9 ± 0.93	0	-	N/A ^4^
Duration of pain control/analgesic effect of drug	468	3.4 ± 0.91	567	3.8 ± 0.73	<0.01
Ease of administration	469	3.5 ± 1.01	566	3.6 ± 0.88	0.54
Short withhold period	467	3.2 ± 1.15	568	3.6 ± 0.95	<0.01
Animal’s ability to feel pain	470	3.6 ± 0.90	569	3.9 ± 0.80	<0.01
Improving safety of the caregiver/operator	468	3.5 ± 1.05	568	3.7 ± 0.94	=0.01
Improved production outcomes	466	3.7 ± 0.97	567	3.7 ± 0.91	0.92
How painful I consider the procedure to be	468	3.5 ± 0.98	569	4.0 ± 0.79	<0.01
Time of onset of drug activity	465	3.4 ± 0.95	569	3.5 ± 0.92	0.10
Request of producer (Veterinarians only)	0	-	568	3.3 ± 0.99	N/A ^4^

^1^*p*-value derived from Mann-Whitney test comparing each factor for producers versus; veterinarians. Statistical significance declared at *p* < 0.05; ^2^ Number of respondents for each factor; ^3^ Food and Administration; ^4^ Not applicable.

## Supplementary Materials

The following are available online at https://www.mdpi.com/2076-2615/11/1/209/s1, Survey S1: Cattle Pain Management Survey.

## References

[1] De Rooij S.J., De Lauwere C., van der Ploeg J.D. (2010). Entrapped in group solidarity? Animal welfare, the ethical positions of farmers and the difficult search for alternatives. J. Environ. Pol. Plan..

[2] Ventura B.A., von Keyserlingk M.A.G., Weary D.M. (2015). Animal welfare concerns and values of stakeholders within the dairy industry. J. Agric. Environ. Ethics..

[3] Winder C.B., LeBlanc S.J., Haley D.B., Lissemore K.D., Godkin M.A., Duffield T.F. (2016). Practices for the disbudding and dehorning of dairy calves by veterinarians and dairy producers in Ontario, Canada. J. Dairy Sci..

[4] Sumner C.L., von Keyserlingk M.A.G., Weary D.M. (2018). Perspectives of farmers and veterinarians concerning dairy cattle welfare. Anim. Front..

[5] Molony V., Kent J., Robertson I. (1995). Assessment of acute and chronic pain after different methods of castration of calves. Appl. Anim. Behav. Sci..

[6] Coetzee J.F. (2011). A review of pain assessment techniques and pharmacological approaches to pain relief after bovine castration: Practical implications for cattle production within the United States. Appl. Anim. Behav. Sci..

[7] Dockweiler J., Coetzee J., Edwards-Callaway L., Bello N., Glynn H., Allen K., Theurer M., Jones M., Miller K., Bergamasco L. (2013). Effect of castration method on neurohormonal and electroencephalographic stress indicators in Holstein calves of different ages. J. Dairy Sci.

[8] Heinrich A., Duffield T., Lissemore K., Millman S. (2010). The effect of meloxicam on behavior and pain sensitivity of dairy calves following cautery dehorning with a local anesthetic. J. Dairy Sci..

[9] Costa J.H., Cantor M.C., Adderley N.A., Neave H.W. (2019). Key animal welfare issues in commercially raised dairy calves: Social environment, nutrition, and painful procedures. Can. J. Anim. Sci..

[10] Adcock S.J., Tucker C.B. (2018). The effect of disbudding age on healing and pain sensitivity in dairy calves. J. Dairy Sci..

[11] Coetzee J.F., Mosher R.A., KuKanich B., Gehring R., Robert B., Reinbold J.B., White B.J. (2012). Pharmacokinetics and effect of intravenous meloxicam in weaned Holstein calves following scoop dehorning without local anesthesia. BMC Vet. Res..

[12] Allen K., Coetzee J., Edwards-Callaway L., Glynn H., Dockweiler J., KuKanich B., Lin H., Wang C., Fraccaro E., Jones M. (2013). The effect of timing of oral meloxicam administration on physiological responses in calves after cautery dehorning with local anesthesia. J. Dairy Sci..

[13] Coetzee J.F., Nutsch A.L., Barbur L.A., Bradburn R.M. (2010). A survey of castration methods and associated livestock management practices performed by bovine veterinarians in the United States. BMC Vet. Res..

[14] Fajt V.R., Wagner S.A., Norby B. (2011). Analgesic drug administration and attitudes about analgesia in cattle among bovine practitioners in the United States. J. Am. Vet..

[15] FOI Freedom of Information Summary. https://animaldrugsatfda.fda.gov/adafda/app/search/public/document/downloadFoi/1944.

[16] Johnstone E.C.S., Coetzee J.F., Pinedo P.J., Edwards-Callaway L. (2021). Survey investigating current attitudes towards use of pain mitigation practices in beef and dairy cattle in the US by veterinarians and producers. J. Am. Vet. Med. Assoc..

[17] Coetzee J.F., Gehring R., Tarus-Sang J., Anderson D.E. (2010). Effect of sub-anesthetic xylazine and ketamine (‘ketamine stun’) administered to calves immediately prior to castration. Vet. Anaesth. Analg..

[18] Gleerup K.B., Andersen P.H., Munksgaard L., Forkman B. (2015). Pain evaluation in dairy cattle. Appl. Anim. Behav. Sci..

[19] Remnant J.G., Tremlett A., Huxley J.N., Hudson C.D. (2017). Clinician attitudes to pain and use of analgesia in cattle: Where are we 10 years on?. Vet. Rec..

[20] Dohoo I., Martin W., Stryhn H. (2009). Veterinary Epidemiologic Research.

[21] Clark B., Stewart G.B., Panzone L.A., Kyriazakis I., Frewer L.J. (2017). A systematic review of public attitudes, perceptions and behaviours towards production diseases associated with farm animal welfare. J. Agric. Environ. Ethics..

[22] Wang J., Gehring R., Baynes R.E., Webb A.I., Whitford C., Payne M.A., Fitzgerald K., Craigmill A.L., Riviere J.E. (2003). Evaluation of the advisory services provided by the Food Animal Residue Avoidance Databank. JAVMA.

[23] Riviere J.E., Tell L.A., Baynes R.E., Vickroy T.W., Gehring R. (2017). Guide to FARAD resources: Historical and future perspectives. JAVMA.

[24] Doing Enough for Disbudding Pain. https://hoards.com/article-27171-doing-enough-for-disbudding-pain.html.

[25] AABP Updates Dehorning Guidelines | Drovers. https://www.drovers.com/news/aabp-updates-dehorning-guidelines.

[26] No Flunixin for Pain Relief. https://hoards.com/blog-19997-no-flunixin-for-pain-relief.html.

[27] FDA Current Drug Shortages. https://www.fda.gov/animal-veterinary/product-safety-information/current-drug-shortages.

[28] APHIS (2014). Dairy 2014 Health and Management Practices on U.S. Dairy Operations. https://www.aphis.usda.gov/animal_health/nahms/dairy/downloads/dairy14/Dairy14_dr_PartIII.pdf.

[29] CROPP Animal Care Program. https://www.farmers.coop/sites/default/files/downloads/cropp_animal_care_standards_010118.pdf.

[30] Spurlock D.M., Stock M.L., Coetzee J.F. (2014). The impact of 3 strategies for incorporating polled genetics into a dairy cattle breeding program on the overall herd genetic merit. J. Dairy Sci..

[31] Rault J.-L., Holyoake T., Coleman G. (2017). Stockperson attitudes toward pig euthanasia. J. Anim. Sci..

[32] Campler M.R., Pairis-Garcia M.D., Rault J.-L., Coleman G., Arruda A.G. (2018). Caretaker attitudes toward swine euthanasia1. Transl. Anim. Sci..

[33] Balzani A., Hanlon A. (2020). Factors that Influence Farmers’ Views on Farm Animal Welfare: A Semi-Systematic Review and Thematic Analysis. Animals.

[34] Huxley J.N., Whay H.R. (2006). Current attitudes of cattle practitioners to pain and the use of analgesics in cattle. Vet. Rec..

[35] Wagner B., Royal K., Park R., Pairis-Garcia M. (2020). Identifying Barriers to Implementing Pain Management for Piglet Castration: A Focus Group of Swine Veterinarians. Animals.

