# Supplementary material for: Producer and Veterinarian Perspectives towards Pain Management Practices in the US Cattle Industry
Source: Animals (Basel). 2021 Jan 16;11(1):209. doi: 10.3390/ani11010209 (PMC7830793; doi:10.3390/ani11010209)
Supplement: Supplementary file 1 [file animals-11-00209-s001.pdf]

Supplementary Materials (S1): **Cattle Pain Management Survey**

Q1 Where is your operation located?

❍ Alabama (1)	❍ Kentucky (18)	❍ Ohio (36)
❍ Alaska (2)	❍ Louisiana (19)	❍ Oklahoma (37)
❍ Arizona (3)	❍ Maine (20)	❍ Oregon (38)
❍ Arkansas (4)	❍ Maryland (21)	❍ Pennsylvania (39)
❍ California (5)	❍ Massachusetts (22)	❍ Puerto Rico (40)
❍ Colorado (6)	❍ Michigan (23)	❍ Rhode Island (41)
❍ Connecticut (7)	❍ Minnesota (24)	❍ South Carolina (42)
❍ Delaware (8)	❍ Mississippi (25)	❍ South Dakota (43)
❍ District of Columbia (9)	❍ Missouri (26)	❍ Tennessee (44)
❍ Florida (10)	❍ Montana (27)	❍ Texas (45)
❍ Georgia (11)	❍ Nebraska (28)	❍ Utah (46)
❍ Hawaii (12)	❍ Nevada (29)	❍ Vermont (47)
❍ Idaho (13)	❍ New Hampshire (30)	❍ Virginia (48)
❍ Illinois (14)	❍ New Jersey (31)	❍ Washington (49)
❍ Indiana (15)	❍ New Mexico (32)	❍ West Virginia (50)
❍ Iowa (16)	❍ New York (33)	❍ Wisconsin (51)
❍ Kansas (17)	❍ North Carolina (34)	❍ Wyoming (52)
	❍ North Dakota (35)	❍ I do not reside in the United States (53)

West: WA (49), OR (38), CA(5), NV(29), UT(46), ID(13), MT(27), WY(52), CO(6)
Southwest: AZ(3), NM(32), TX(45), OK(37),
Midwest: ND(35), SD(43), NE(28), KS(17), MN(24), IA(16), MO(26), WI(51), IL(14), IN(15), MI(23), OH(36)
Southeast: AR(4), LA(19), MS(25), AL(1), TN(44), KY(18), GA(11), FL(10), SC(42), NC(34), WV(50), VA(48), DC(9), MD(21), DE(8)
Northeast: NJ(31), PA(39), RI(41), CT(7), NY(33), MA(22), VT(47), NH(30), ME(20)

Q2 Are you?

- ❍ Male (1)
- ❍ Female (2)

Q3 Which of the following best describes your involvement with the cattle industry? (Select all that apply.)

- ❑ Producer (beef or dairy) (3)
- ❑ Veterinarian (2)
- ❑ Other (please specify): (1) __= Both vet and producer____

Q7 How old are you?

- ❍ Under 20 (1)
- ❍ 21 to 30 (2)
- ❍ 31 to 40 (3)
- ❍ 41 to 50 (4)
- ❍ 51 to 60 (5)
- ❍ 61 to 70 (6)
- ❍ 1 (7)

Display This Question: If Q3 = Producer (beef or dairy)

Q9 What is the highest degree or level of school you have completed? If currently enrolled, highest degree received.

- ❍ Did not complete high school (1)
- ❍ High School (2)
- ❍ Some College, no degree (3)
- ❍ Trade/technical/vocational training (4)
- ❍ Associate Degree (5)
- ❍ Bachelor’s Degree (6)
- ❍ Master’s Degree (7)
- ❍ Professional Degree (JD, MD) (8)
- ❍ Doctorate Degree (PhD) (9)

Display This Question: If Q3 = Veterinarian

Q10 When did you graduate from veterinary school?

- ❍ Prior to 1970 (1)
- ❍ 1970-1980 (2)
- ❍ 1981-1990 (3)
- ❍ 1991-2000 (4)
- ❍ 2001-2010 (5)
- ❍ 2011-2018 (6)

Q21 Which pain relief drugs (analgesics) do you have knowledge of and feel comfortable using in your operation or practice? (Select all that apply.)

- ❑ Lidocaine (1)
- ❑ Oral Meloxicam (2)
- ❑ Meloxicam Injection (Metacam® Injection) (3)
- ❑ Flunixin (e.g. Banamine®) Injection (4)
- ❑ Flunixin (e.g. Banamine®) pour-on (5)
- ❑ Aspirin (6)
- ❑ Phenylbutazone (7)
- ❑ Ketoprofen (Anafen® Injection) (8)
- ❑ Other (please specify): (9) ____
- ❑ None of these (10)

Q25 How important are the following factors in impacting your decision to use an analgesic drug in adult cattle and calves?

	Not at all important (1)	Slightly important (2)	Moderately important (3)	Very important (4)	Extremely important (5)
FDA Approval status (1)	❍	❍	❍	❍	❍
Cost of the drug (2)	❍	❍	❍	❍	❍
Recommendation of veterinarian (Producers only)	❍	❍	❍	❍	❍
Lack of sedative effect (4)	❍	❍	❍	❍	❍
Duration of Pain Control/ Analgesic effect of drug (5)	❍	❍	❍	❍	❍
Ease of administration (6)	❍	❍	❍	❍	❍
Short Withhold Period (7)	❍	❍	❍	❍	❍
Animal’s ability to feel pain (8)	❍	❍	❍	❍	❍
Improving Safety of the caregiver/ operator (9)	❍	❍	❍	❍	❍
Improved production outcomes (10)	❍	❍	❍	❍	❍
How painful I consider the procedure to be (11)	❍	❍	❍	❍	❍
Time of onset of drug activity (12)	❍	❍	❍	❍	❍
Request of producer (Veterinarians only)	❍	❍	❍	❍	❍

Q30 How has your use of analgesics changed in the last 10 years?

- ❍ Increased use (1)
- ❍ Stayed the same (2)
- ❍ Decreased use (3)

Display This Question: If Q30 = Increased use

Q31 Why has your use of analgesics increased? (Select all that apply.)

- ❑ New evidence of analgesic effectiveness (1)
- ❑ Requirement of a quality assurance program (2)
- ❑ Decreased prices for analgesics (3)
- ❑ Change in your perception of pain in cattle (4)
- ❑ Changing farmer or veterinarian attitudes (5)
- ❑ Change in practice or operation protocols (6)
- ❑ Influence from colleagues/fellow producers (7)
- ❑ Mandated by a retailer or packer (8)
- ❑ Maintain consumer confidence in livestock production practices (9)
- ❑ Cattle that receive analgesia look better than cattle that don’t (10)
- ❑ Cattle that receive analgesia have improved health and performance (11)

Display This Question: If Q30 = Decreased use

Q32 Why has your use of analgesics decreased? (Select all that apply.)

- ❑ Currently available analgesic drugs are not effective at reducing pain (1)
- ❑ Currently available analgesic drugs are inconvenient to administer (2)
- ❑ Currently available analgesic drugs do not last long enough after 1 dose to justify their use (3)
- ❑ Currently available analgesic drugs are too expensive (4)
- ❑ I do not know the meat and milk withhold periods for the analgesic drugs (5)
- ❑ Currently available drugs do not improve health and performance (6)
- ❑ I am not comfortable using an analgesic unless it has been approved by FDA (7)

Q37 Do you consider that your knowledge about recognizing and treating pain in adult cattle and calves is adequate?

- ❍ Yes (1)
- ❍ No (2)

Q38 Where do you feel you have obtained most of your knowledge about recognizing and treating pain in adult cattle and calves? **Remnant adapted

- ❍ FFA/ 4-H training (1)
- ❍ College classes (2)
- ❍ Journals / articles (3)
- ❍ Continuing education (4)
- ❍ Personal Experience (5)
- ❍ Online training modules (6)
- ❍ Commercial literature / data sheets (7)
- ❍ Other (please specify): (8) ____

Display This Question: If Q3 = Veterinarian

Q39 Does the type of illness or procedure affect the likelihood of using a specific analgesic drug in the cattle you treat?

- ❍ Yes (1)
- ❍ No (2)

Display This Question: If Q39 = No

Q40 How likely are you to consider using the following analgesic drugs in cattle?

	Extremely unlikely (1)	Somewhat unlikely (2)	Neither likely nor unlikely (3)	Somewhat likely (4)	Extremely likely (5)
Lidocaine (1)	❍	❍	❍	❍	❍
Oral Meloxicam (2)	❍	❍	❍	❍	❍
Meloxicam Injection (3)	❍	❍	❍	❍	❍
Flunixin (Banamine) Injection (4)	❍	❍	❍	❍	❍
Flunixin (Banamine) pour-on (5)	❍	❍	❍	❍	❍
Aspirin (6)	❍	❍	❍	❍	❍
Phenylbutazone (7)	❍	❍	❍	❍	❍
Ketoprofen (8)	❍	❍	❍	❍	❍
Other (please specify): (9)	❍	❍	❍	❍	❍
